# Identification of a new European rabbit IgA with a serine-rich hinge region

**DOI:** 10.1371/journal.pone.0201567

**Published:** 2018-08-08

**Authors:** Ana Pinheiro, Patricia de Sousa-Pereira, Tanja Strive, Katherine L. Knight, Jenny M. Woof, Pedro J. Esteves, Joana Abrantes

**Affiliations:** 1 CIBIO Centro de Investigação em Biodiversidade e Recursos Genéticos, InBio Laboratório Associado, Universidade do Porto, Campus Agrário de Vairão, Vairão, Portugal; 2 Departamento de Biologia, Faculdade de Ciências, Universidade do Porto, Porto, Portugal; 3 Max von Pettenkofer-Institute for Virology, Ludwig-Maximilians-University Munich, Munich, Germany; 4 Commonwealth Scientific and Industrial Research Organization, Canberra, ACT, Australia; 5 Department of Microbiology and Immunology, Stritch School of Medicine, Loyola University Chicago, Maywood, Illinois, United States of America; 6 Cell Signalling and Immunology, School of Life Sciences, University of Dundee, Dundee, United Kingdom; 7 Centro de Investigação em Tecnologias da Saúde, IPSN, CESPU, Gandra, Portugal; Chang Gung University, TAIWAN

## Abstract

In mammals, the most striking IgA system belongs to Lagomorpha. Indeed, 14 IgA subclasses have been identified in European rabbits, 11 of which are expressed. In contrast, most other mammals have only one IgA, or in the case of hominoids, two IgA subclasses. Characteristic features of the mammalian IgA subclasses are the length and amino acid sequence of their hinge regions, which are often rich in Pro, Ser and Thr residues and may also carry Cys residues. Here, we describe a new IgA that was expressed in New Zealand White domestic rabbits of *IGHV*a1 allotype. This IgA has an extended hinge region containing an intriguing stretch of nine consecutive Ser residues and no Pro or Thr residues, a motif exclusive to this new rabbit IgA. Considering the amino acid properties, this hinge motif may present some advantage over the common IgA hinge by affording novel functional capabilities. We also sequenced for the first time the IgA14 CH2 and CH3 domains and showed that IgA14 and IgA3 are expressed.

## Introduction

The European rabbit (*Oryctolagus cuniculus)* has the most complex immunoglobulin A (IgA) system observed in mammals, with 13 IGHA genes (Cα) encoding the heavy chain constant regions for 13 IgA subclasses [1]; a fourteenth *IGHA* was partially described [2]. Of these, 11 IgA subclasses are expressed and show differential tissue expression [3]. The Cα3 and Cα8 genes have defective Iα promoter regions and as a result are not expressed *in vivo* [4]. This complex system is even more remarkable and intriguing given that mice [5] and most other mammals have one IGHA gene, coding for one IgA isotype, and hominoid primates (with the exception of orangutan) have two IGHA genes that code for the IgA1 and IgA2 subclasses [6]. These two IGHA genes arose by gene duplication in a common hominoid primate ancestor [6]. Similarly, the multiplication of the rabbit Cα genes seems to have begun at least 35 million years ago in an ancestral Lagomorph since multiple IgA copies have been found for *Sylvilagus*, *Lepus*, *Pentalagus* and *Ochotona* genera [1, 7].

Characteristic features of the mammalian IgA subclasses are the length and amino acid sequence of their hinge regions [1, 8]. Human IgA1 has an extended hinge which includes a 16 amino acid insertion rich in Pro, Ser and Thr residues not present in the short, proline rich, IgA2 hinge. The Ser and Thr residues of the human IgA1 hinge may carry three to six O-linked oligosaccharides [9–12] which may confer some protection to proteolytic cleavage [8] and are likely to affect the hinge structure decreasing its conformational variability [13]. Similar to the IgA hinge regions of most mammals, the rabbit IgA hinge regions are rich in Pro, Ser and Thr residues, but also carry Cys residues, absent in human IgA hinge regions. Most of the European rabbit IgA subclasses also feature an extended hinge (IgA2, IgA3, IgA4, IgA5, IgA6, IgA8, IgA10, IgA13 and IgA14) [1].

IgA is the most abundantly secreted immunoglobulin isotype in mammals, and the predominant isotype in mucosal tissues and external secretions. IgA provides a major line of defense against pathogens and plays a key role in the maintenance of the commensal microbiota in the intestinal tract [14]. Evidence that this key role of IgA in immune protection extends to rabbits includes the finding that an important IgA response is observed in rabbits that survive rabbit hemorrhagic disease virus infection [15, 16], a highly lethal disease that has been decimating European rabbit populations since the 1980’s [17, 18]. Similarly, rabbits infected with the non-pathogenic calicivirus RCV-A1, which is related to RHDV but causes a non-clinical infection of the small intestine [19, 20], mount a strong IgA response following infection [21].

Much of the *IGHA* locus containing the known 13 Cα genes in the rabbit genome has been mapped [1], but the organization of these genes within the locus remains incomplete [1,22]. We hypothesized that additional Cα genes may reside in the rabbit genome, and in this study we sequenced mRNA encoding IgA α-chains from New Zealand White domestic rabbits of *IGHVa1* allotype. We report the identification of mRNA encoding a new IgA heavy chain with a unique hinge region. We also determined the complete sequence of the previously identified, partially sequenced, *Cα14*.

## Material and methods

### Samples, PCR-amplification and sequencing of expressed and genomic IgA α-chain

Samples of small intestine from four New Zealand rabbits (*Oryctolagus cuniculus cuniculus*) were collected immediately post-mortem and preserved in RNAlater. These rabbits were obtained from the domestic rabbit breeding colony at CSIRO Black Mountain. All procedures involving animals were carried out in accordance with the Australian Code for the Care and Use of Animals for Scientific Purposes and were approved by the CSIRO Ecosystem Sciences Animal Ethics Committee (Licence # ESAEC 13–11). For euthanasia, rabbits were first sedated by intramuscular injection of 30 mg/kg ketamine and 5 mg/kg Xylazine, followed by intravenous injection of 165 mg/kg sodium pentobarbitone. Total RNA was extracted using the RNeasy Mini Kit (Qiagen, Hilden, Germany) according to the manufacturer’s protocol, followed by first-strand complementary cDNA synthesis with the SuperScriptTM III Reverse Transcriptase Kit (Invitrogen) using 1μg RNA. To PCR amplify rabbit Cα transcripts, primers were designed in conserved regions of the CDS for all known Cα genes in rabbit: Calpha_ocFw (5’ CTGCCTGATCCRGGGCTTC 3’) in the CH1 domain and Calpha_ocRv (5’ CCACGACCACAGACACGTTG 3’) in the CH3 domain. The PCR was performed using the PCR Master Mix (Promega) with annealing temperature of 59° C for 45 seconds and 1 minute extension, for 35 cycles. PCR products were purified (NucleoSpin Gel and PCR Clean-up kit, Macherey-Nagel, Germany) and cloned into the pGEM-T Easy vector system II (Promega, Madison, WI, USA). For each rabbit, sixteen clones were selected. Sequencing was performed on an ABI PRISM 310 Genetic Analyser (PE Applied Biosystems).

Genomic DNA was extracted from the tissue samples used previously for RNA extraction, using the EasySpin Genomic DNA Minipreps Tissue Kit (Citomed) according to the manufacturer’s instructions. The exact same procedure described above was used to amplify the genomic region of the new Cα gene and determine the intron sequence.

### Phylogenetic analyses

The obtained sequences were aligned with Rabbit IgA1 to IgA14 sequences using Multiple Sequence Comparison by Log-Expectation (MUSCLE) available at http://www.ebi.ac.uk/ [23] and translated using BioEdit [24]. Rabbit IgA1 to IgA14 sequences were obtained from GenBank (http://www.ncbi.nlm.nih.gov/genbank/) under the following accession numbers: X51647, X82108 to X82119, AF314407 and AY386696. MEGA6.0 software [25] was used to construct a phylogenetic tree and to calculate genetic distances. The phylogenetic tree was constructed using the maximum likelihood (ML) method and the GTR+G model of nucleotide substitution, determined as the optimal in the same software according to the lowest AICc (corrected Akaike information criterion) value. Node support was determined from 1000 bootstrap replicate ML trees. Genetic distances between rabbit *IGHA*s CDS and intronic regions were calculated using the p-distance method and pairwise deletion of gaps. Standard errors estimates were obtained after 500 bootstraps procedure.

### Rabbit *VH* allotype determination

The *VH* allotype of the analyzed rabbits was determined by sequencing the expressed VDJ genes. Total RNA extraction for spleen tissues was as described above followed by cDNA synthesis with the iScript cDNA Kit (Bio-Rad, California, USA) using 1μg RNA. Rearranged VDJ genes were PCR-amplified using primers VH (5′GGAGACTGGGCTGCGCTGGCTTCTCCTGGT3′; [26]) and JH2 (5’TGAGGAGACGGTGACCAGGGTGCCT3’; [27]). PCR amplification conditions were as follows: 4 min at 95°C followed by 35 cycles at 95°C (45 s), 62°C (45 s) and 72°C (60 s), with a final extension at 72°C (5 min). PCR products were purified (MinElute PCR Purification Kit, Qiagen, Hilden, Germany) and cloned as described above.

The 96 VDJ genes obtained for the four analysed rabbits were aligned with available sequences for European rabbit VDJ genes taken from GenBank, specifically rabbit germline VH1 and VH4 genes of a1, a2 and a3 allotypes (accession numbers M93171, M93181, M93172, M93182, M93173, L03846), rabbit cDNA VH gene sequences representative of allotypes a1, a2, a3, a4.1 and a4.2 (AF029933, AF029934, AF029938, AF029940, L03849, L03851, L03853, L03856, AF029923, AF098235, AF264434, AF264435, AY207979- AY207982, AY208042, AY208047, AY207967) and rabbit germline and rabbit VH gene sequences of VHx, VHy, and VHz (L03846, M19706, L03890, L03874, AF264469, AY207986, AY208045, AY208006). Neighbor-joining phylogenetic analyses were conducted using the p-distance method and pairwise deletion of gaps to compute evolutionary distances in MEGA6.0 software [25]. Of the 96 obtained genes 92 *VDJ* sequences classified as encoding the *VH*a1 allotype and the remaining four VDJ genes as encoding *VH*n. We conclude that all four analyzed rabbits have *VH*a1 allotype and are homozygous for the *a*^*1*^ allele (*a*^*1*^*/a*^*1*^).

## Results

To search for new IgA molecules, we sequenced IgA heavy chains from cDNA samples of New Zealand White domestic rabbits. We found cDNA encoding Cα genes of IgA1, IgA2, IgA3, IgA4, IgA5, IgA9, IgA10 and IgA12 (accession numbers: MH120859 to MH120866) as well as two unidentified Cα’s: one being expressed in all four analyzed animals and the other found in two animals. The sequences obtained for IgA1, IgA2, IgA4, IgA9, IgA10 and IgA12 are nearly identical, >97% sequence identity, to those published for the G and F-I haplotypes (accession numbers: X51647, X82108, X82110, X82115, X82116, X82118 and AY386696). The sequence obtained for IgA5 has 100% sequence identity to IgA5b of the F-I haplotype (AY386696) and only 96% identity to IgA5 of the G haplotype (X82111). The sequence obtained for IgA3 has 99% identity to the sequence of the published IgA3 sequence (X82109); however this was a partial sequence, spanning CH1 and hinge region domains.

**Fig 1:**
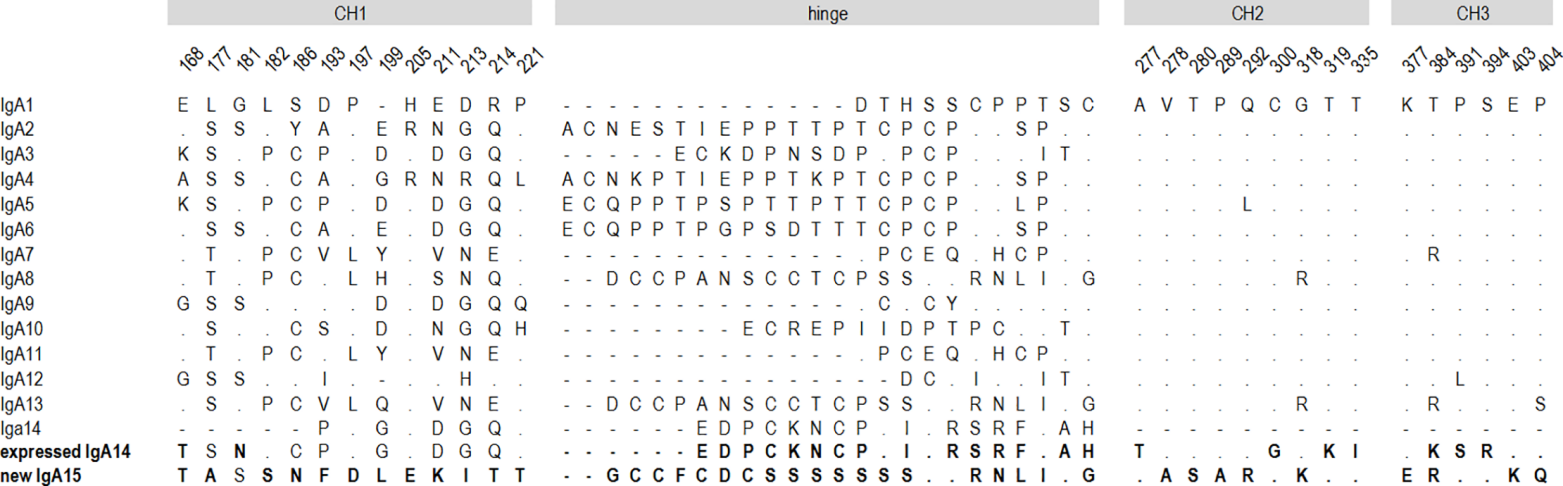
Amino acids sequence alignment of European rabbit 15 IgA’s. The entire hinge region sequences are depicted. For the remaining constant region domains, only the positions for which IgA15 and IgA14 Cα chains have unique amino acid residues are shown. Human IgA1 numbering for these positions is shown above. Dots (.) represent identity with the uppermost sequence, dashes (-) stand for gaps in the alignment. IgA6 is presumably an allele to IgA1 [29]. GenBank accession numbers for IgA1 to IgA14 are X51647, X82108 to X82119, and AF314407. Accession number for the new IgA15 and expressed IgA14 are MH120868 and MH120867.

**Fig 2:**
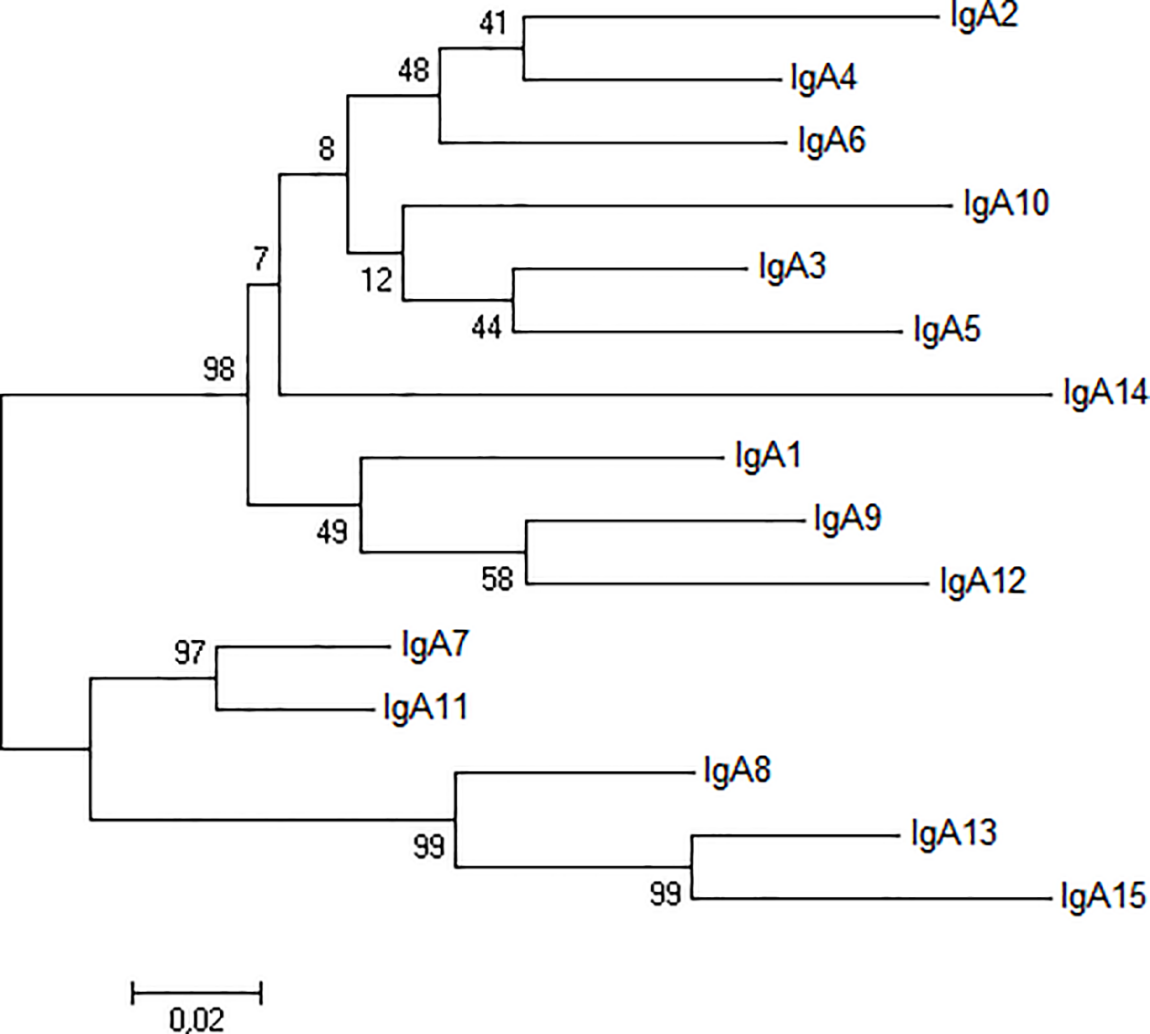
Phylogenetic tree of European rabbit 15 IgA’s. The ML phylogenetic tree of European rabbit 15 IgA’s is shown. Sequences used include IgA1 to IgA13 (GenBank accession numbers X51647 and X82108 to X82119), the expressed IgA14 and the new IgA15 (GenBank accession numbers MH120867 and MH120868).

Of the unidentified Cα genes we found, the ubiquitously expressed one encodes a hinge region distinct from all described rabbit Cα hinge regions (Fig 1, S1 Fig), indicating that this transcript encodes a new rabbit IgA Cα molecule. We designate this as the heavy chain for a new isotype, IgA15 (accession number MH120868). To analyze the relationship between this new IgA15 and the remaining rabbit IgA’s a phylogenetic tree was constructed. The obtained ML tree shows three well-supported clusters (Fig 2): the largest cluster encompasses IgA1-IgA6, IgA9, IgA10, IgA12 and IgA14 (98 bootstrap value), a second group clusters IgA7 and IgA11 (99 bootstrap value) and the third cluster includes IgA8, IgA13 and the new IgA15 (99 bootstrap value). The new IgA15 is, thus, phylogenetically closer to IgA8 and IgA13. The obtained tree topology suggests that at least three duplication events are at the origin of the European rabbit 15 IgA’s. The genetic distance between the Cα of IgA15 and other rabbit IgA Cα genes (0.09–0.38; Table 1) is similar to that observed between the other rabbit IgA’s (0.05–0.38; Table 1); the highest similarity for IgA15 is with IgA8 and IgA13 (Table 1). As has been observed for rabbit and other mammalian *IGHA* [1, 28], the CH1 domain encoded by Cα15 is the most extensively diversified (genetic distances range 0.14–0.33); CH2 and CH3 domains (genetic distances range 0.05–0.17 and 0.06–0.13, respectively) are more conserved. The high similarity observed between the IgA obtained in this work and the IgA described by Ros et al. [22] suggests that the rabbits studied in this work most likely belong to the I haplotype. Given this, we cannot ascertain if the newly described Cα15 constitutes a new locus, or an allele of another Cα gene. In fact, from the similarity to IgA8 and IgA13 (see Table 1), Cα15 may be allelic to one of these known loci.

**Table 1:** Genetic distances between European rabbit IgA subclasses nucleotide sequences.

IgA subclass	IgA1	IgA2	IgA3	IgA4	IgA5	IgA6	IgA7	IgA8	IgA9	IgA10	IgA11	IgA12	IgA13	IgA14	IgA15
**IgA1**															
**IgA2**	0.14														
**IgA3**	0.12	0.15													
**IgA4**	0.13	0.12	0.13												
**IgA5**	0.15	0.14	0.09	0.13											
**IgA6**	0.13	0.12	0.15	0.13	0.13										
**IgA7**	0.12	0.17	0.14	0.14	0.15	0.15									
**IgA8**	0.14	0.19	0.15	0.19	0.19	0.21	0.11								
**IgA9**	0.11	0.13	0.09	0.14	0.14	0.14	0.15	0.15							
**IgA10**	0.14	0.17	0.13	0.15	0.15	0.14	0.14	0.17	0.13						
**IgA11**	0.13	0.17	0.13	0.15	0.15	0.15	0.05	0.10	0.14	0.13					
**IgA12**	0.10	0.15	0.12	0.14	0.15	0.14	0.15	0.17	0.09	0.13	0.15				
**IgA13**	0.16	0.19	0.17	0.19	0.19	0.20	0.13	0.09	0.17	0.18	0.13	0.19			
**IgA14**	0.25	0.28	0.31	0.25	0.31	0.32	0.38	0.33	0.28	0.29	0.38	0.29	0.33		
**IgA15**	0.17	0.20	0.17	0.19	0.20	0.21	0.15	0.12	0.17	0.19	0.15	0.18	0.09	0.38	
**expressed IgA14**	0.13	0.14	0.13	0.13	0.16	0.15	0.16	0.16	0.13	0.16	0.16	0.14	0.16	0.02	0.16

Genbank accession numbers for IgA1 to IgA14 sequences are X51647, X82108 to X82119 and AF314407. Genbank accession numbers for IgA15 and expressed IgA14 are MH120868 and MH120867.

Genetic distances between sequences, calculated using the p-distance, are shown below the diagonal. All ambiguous positions were removed for each sequence pair.

To confirm that this new IgA transcript is present in the germline we undertook its PCR amplification from genomic DNA. The resulting new genomic Cα shows the typical IGHA gene structure in which each one of the constant domains–CH1, CH2 and CH3—is encoded by a separate exon (GenBank accession number: KY131968). The hinge region is encoded along with the CH2 domain in exon 2, in keeping with other *IGHA* genes. Intron sequences are unique: divergence from other *IGHA* intronic regions ranges from 7% to 38%, in line with the divergence found between other Cα gene introns (2% to 41%; data not shown).

The newly described IgA15 α-chain has 19 unique amino acid residues, when compared to other European rabbit IgA subclasses: 11 of these positions are found in the CH1 domain, five in the CH2 domain and three in the CH3 domain (Fig 1, S1 Fig), in accordance with the estimated nucleotide genetic distances. The hinge presents a unique sequence. Indeed, the IgA15 Cα-chain has an extended hinge with an intriguing stretch of nine consecutive Ser residues and it contains no Pro or Thr residues, unlike the other rabbit IgA hinge regions (Fig 1, S1 Fig). Comparisons to mammalian and other vertebrate immunoglobulins, show that this motif is exclusive to this new rabbit IgA.

The other unidentified Cα transcript that we found encoded a hinge region identical to the IgA14 hinge region, and was nearly identical (98% similarity; Table 1) to the partial IgA14 sequence, spanning mid CH1 to early CH2 domain (accession number AF314407). We conclude that this gene encodes the complete sequence for the IgA14 Cα chain (accession number MH120867). The genetic distance between the Cα of the full IgA14 and other rabbit IgA’s (0.13–0.16; Table 1) is within the range observed between rabbit IgA’s (0.05–0.38; Table 1). As has been observed for rabbit and other mammalian *IGHA* [1, 28], the CH1 domain encoded by Cα14 is the most extensively diversified (genetic distances range 0.14–0.31) whereas the CH2 and CH3 domains (genetic distances range 0.09–0.14 and 0.08–0.12, respectively) are more conserved. Our IgA14 sequence has nine unique amino acid residues: two in the CH1 domain, four in the CH2 domain and three in the CH3 domain.

## Discussion

Members of the order Lagomorpha are the only mammals known to have more than two *IGHA* genes [1, 2]. Understanding how the rabbit mucosal immune system has adapted to this large number of IgA subclasses may open new therapeutic possibilities [30, 31]. In this study we describe the CH2 and CH3 domains of IgA14 as well as an additional IgA being expressed in domestic European rabbits. Despite having used primers designed to bind conserved regions of all rabbit IgA subclasses, and hence putatively able to amplify all of the known 14 subclasses, we found only ten rabbit IgA’s encoded by the total of 64 clones examined. The absence of the other four IgA subclasses was likely due to differences in expression level of the different IgA’s. IgA expression in the rabbit is regulated by several elements. Among these is a negative regulatory element (NRE) associated with rabbit germline Cα genes that negatively regulates the Iα promoter and hs1,2 enhancement of this promoter. The activity of this Cα-NRE may contribute to the differences in expression level of the multiple IGHA genes [32].Testing the expression of European rabbit IgA subclasses in several mucosal tissues Spieker-Polet et al [3] found some subclasses were expressed at a much higher level than others, with some inter-individual variation. The subclasses identified in this study may well be expressed at higher levels than others in the individuals analysed, and thus were more easily sequenced.

IgA14 and IgA15 were both expressed in New Zealand White domestic breed animals, the common laboratory rabbit for which the IgA genes have been sequenced previously [1, 2, 22], but our sequences represent the first study of rabbits with the *VH*1a1 allotype. Sequencing of expressed VDJ genes for wild European rabbits identified a fourth *VH*a lineage, unobserved in domestic breed animals [33]. Similarly, sequencing the complete *IGHG* for wild European rabbits identified new polymorphic positions [34, 35]. The European rabbit domestic breeds originated approximately 1500 years ago [36] from French wild *O*. *cuniculus cuniculus*, and have been shown to represent a subset of these populations with decreased genetic diversity compared to wild European rabbit populations from the Iberian Peninsula [33, 34, 37–39], where the species originated [40]. Accordingly, it is likely that differences in *IGHA* sequence and total number of copies may be found in wild European rabbit populations. Kingzette et al. [41] suggested differences in *IGHA* subclasses could exist between different European rabbit *IGH* haplotypes, and later, Volgina et al. [2] identified the fourteenth IgA by partially sequencing *IGHA* from a rabbit of a different haplotype than those for which the 13 IgA’s had been sequenced originally (E haplotype, *VH*1a2 allotype, and G haplotype, *VH*1a3 allotype, respectively). More recently, sequencing of *VH*1a2 allotype rabbits did not identify other *IGHA* [22, 42]. Interestingly, the IgA3 that is not expressed in the G haplotype due to a defect in the promoter was found in this study, most likely due to differences in the promoter region between haplotypes.

Using Southern blot analysis Burnett et al [1] showed that *Sylvilagus*, *Lepus*, *Pentalagus* and *Ochotona* genera have multiple IgA copies and, hence, the duplication of the IGHA genes began in an ancestral Lagomorph at least 35 million years ago. Several hypotheses have been put forward to explain this extraordinary expansion of the IgA family in the European rabbit. Burnett et al [1] argued that bacterial IgA proteases may have influenced the duplication of the IGHA genes in lagomorphs as the different IgA subclasses must show different susceptibilities to proteolytic cleavage. In fact, the mammalian IgA’s have been suggested to be evolving under pathogen pressure [28, 43], and hence, pathogens may exert a similar pressure on the IgA of Lagomorphs. An alternative hypothesis to explain the high number of IgA copies is that the duplication of IgA arose as a compensatory mechanism to the absence of multiple IgG subclasses in the rabbit, since most other studied mammals have multiple IgG subclasses [44–50].

The IgA15 α-chain has a unique hinge region with a sequence of nine consecutive Ser residues, unprecedentedly observed in mammalian immunoglobulins. *Bona fide* Igs are characteristic of jawed vertebrates [51] and the most ancient mucosal Ig is teleost fish IgT/Z, being the main Ig induced in gut, skin and gill mucosa of bony fish [52]. Other mucosal Igs are the orthologous amphibian IgX and avian, reptilian and mammalian IgA. Among these mucosal Igs, mammalian IgA is the only which presents a hinge region. Despite its high sequence similarity to avian and reptilian IgA, mammalian IgA has lost the second of the four constant domains of precursor IgA and gained a hinge [51]. Other Ig isotypes bearing a hinge region are fish IgH, amphibian IgF, platypus IgY/O and mammalian IgD and IgG [53] but none of these carry a motif resembling the stretch of nine consecutive serines described here. These Ig isotypes constitute three phylogenetically unrelated groups and so hinge regions seem to have arisen independently three times [53]. The flexibility between the two Fab arms and the Fc region introduced by the hinge region thus seems to be functionally relevant and mutations enhancing this function should be favored. Single amino acid repeats of serine, and other amino acids such as glutamine, glutamic acid, glycine and alanine, have been detected in a wide range of proteins [54], and are thought to have arisen through strand slippage or other replication errors. The structure adopted by such repeats appears to depend on the context within the particular protein. In this case, the stretch of serine residues is likely to contribute to the hinge’s role as a spacer between the Fab and Fc regions. Mammalian IgA hinges are commonly rich in Pro, Ser and Thr residues and also carry Cys residues, but Pro and Thr residues are absent from the *Cα*15 hinge region. Ser is a small and slightly polar amino acid being commonly found in tight turns on the protein surface where it can form a hydrogen bond with the protein backbone, mirroring Pro [55]. Along with Thr residues, Ser residues can also carry O-glycans. These can affect the hinge flexibility, protect the extended hinge regions from bacterial proteases and can further bind pathogens [56]. It would be interesting to determine if the hinge region of IgA15 binds jacalin, a lectin that has been shown to bind O-glycosylated rabbit IgA [57]. One advantage of the IgA15 hinge region may be of increased resistance to proteases. It’s length and amino acid sequence, which mainly characterize the different mammalian IgA subclasses, affect IgA subclass susceptibility to cleavage by bacterial proteases [1,8]. The human and great apes IgA1, featuring an extended hinge, is susceptible to proteolytic cleavage while IgA2 is resistant. IgA1 proteases are post proline endopeptidases that cleave at either Pro-Ser (type 1 enzymes) or Pro-Thr (type 2 enzymes) peptide bonds within the IgA1 hinge region. The amino acid sequence of the new IgA lacks Pro residues which may render it more resistant to proteolytic cleavage while possibly retaining the advantages of an extended hinge such as increased flexibility and potential for cross-linking antigens on the surface of bacteria and other pathogens [58]. Thus, the *Cα*15 hinge region with a stretch of nine Ser residues and no Pro or Thr residues may present some advantage, such as extended reach between the Fab arms or enhanced flexibility, over the common IgA hinge; alternatively, it may simply represent an efficient substitution. Functional studies are needed to answer this question.

## Supporting information

S1 Fig Amino acid sequence alignment of European rabbit 15 IgA Cα chains. Dots (.) represent identity with the uppermost sequence, dashes (-) stand for gaps in the alignment. IgA6 is presumably an allele to IgA1 [26]. GenBank accession numbers are in brackets.
(XLS)
